# Ethanol Preference and Drinking Behavior Are Controlled by RNA Editing in the Nucleus Accumbens

**DOI:** 10.3389/fnbeh.2018.00331

**Published:** 2019-01-15

**Authors:** Takahira Shirahase, Yoshihisa Watanabe, Atsushi Tsujimura, Shin Kwak, Toshiro Yamamoto, Narisato Kanamura, Masaki Tanaka

**Affiliations:** ^1^Department of Basic Geriatrics, Graduate School of Medical Science, Kyoto Prefectural University of Medicine, Kyoto, Japan; ^2^Department of Anatomy and Neurobiology, Graduate School of Medical Science, Kyoto Prefectural University of Medicine, Kyoto, Japan; ^3^Department of Dental Medicine, Graduate School of Medical Science, Kyoto Prefectural University of Medicine, Kyoto, Japan; ^4^Graduate School of Medicine and Faculty of Medicine, University of Tokyo, Tokyo, Japan

**Keywords:** RNA editing, ethanol preference, ethanol drinking behavior, ADAR2, nucleus accumbens

## Abstract

RNA editing plays critical roles in normal brain function, and alteration of its activity causes various disorders. We previously found that chronic consumption of ethanol was associated with increased levels of RNA editing of serotonin 2C receptor in the nucleus accumbens (NAc). However, it remains unknown whether RNA editing in the NAc modulates alcohol addiction through the brain reward system. To investigate the involvement of NAc RNA editing in alcohol addiction, we generated NAc-specific knockout mice of the double-stranded RNA-specific adenosine deaminase *ADAR2* using AAV-GFP/Cre and conducted a battery of behavioral tests including anxiety- and depression-like behaviors. In addition, NAc-specific *ADAR2* knockout mice were exposed to ethanol vapor for 20 days, followed by ethanol-drinking and conditioned place preference (CPP) tests. NAc-specific *ADAR2* knockout mice showed a significant decrease in locomotor activity in the open field test although they did not develop anxiety- and depression-like behaviors. In addition, the enhancements of ethanol intake and ethanol preference that are usually observed after chronic ethanol vapor exposure were significantly reduced in these mice. These results suggest that ADAR2-mediated RNA editing in the NAc is involved in determination of alcohol preference after chronic alcohol consumption.

## Introduction

Adenosine deaminases acting on RNA (ADAR1 and 2) modify adenosine to inosine in double-stranded RNA, a process called RNA editing (Nishikura, 2016). RNA editing is involved in neuronal functions including synaptic plasticity and neuronal development (Behm and Öhman, 2016). The precursor mRNAs of several neuroreceptors such as serotonin 2C receptor (5-HT_2C_R), glutamate receptor A2 (GluA2), and γ-aminobutyric acid type A receptor (GABA_A_) undergo RNA editing, which modulates synaptic transmission (Behm and Öhman, 2016). 5-HT_2C_R mRNA is edited at five positions by ADAR1 and ADAR2, substituting three amino acids in the second intracellular loop region (Burns et al., 1997; Niswender et al., 1998). Edited 5HT_2C_R isoforms show reductions in 5-HT potency, agonist binding affinity, constitutive activity and G-protein coupling activity compared with non-edited isoforms (Burns et al., 1997; Fitzgerald et al., 1999; Niswender et al., 1999; Wang et al., 2000; Gurevich et al., 2002). In mice, alteration in expression of 5HT_2C_R isoforms causes abnormal emotional, feeding and alcohol-drinking behaviors (Kawahara et al., 2008; Mombereau et al., 2010; Martin et al., 2013; Watanabe et al., 2014; Aoki et al., 2016). AMPA receptor subunit GluA2 is also edited at the Q/R site by ADAR2, resulting in reduced Ca^2+^ permeability (Sommer et al., 1991). Impairment of RNA editing in motor neurons of the anterior horn causes neurodegeneration and leads to development of amyotrophic lateral sclerosis (Kawahara et al., 2004; Yamashita and Kwak, 2014).

Accumbal RNA editing is involved in alcohol-drinking, cocaine-seeking and despair behaviors (Watanabe et al., 2014; Schmidt et al., 2015; Aoki et al., 2016). Chronic alcohol exposure causes enhanced 5-HT_2C_R RNA editing following an increase in ADAR1 and ADAR2 expression in the nucleus accumbens (NAc) and dorsal raphe nucleus (DRN) of C57BL/6J mice (Watanabe et al., 2014). As a result of alteration in expression of 5-HT_2C_R isoforms, voluntary alcohol intake is enhanced (Watanabe et al., 2014). Moreover, mice that exclusively express the unedited (INI) isoform of 5-HT_2C_R mRNA on a C57BL/6J background exhibit a reduction in neuropeptide Y (NPY) expression in accumbal neurons and elevated despair behavior compared with wild-type mice (Aoki et al., 2016). In addition, GluA2 RNA editing at the Q/R site in the NAc shell is reduced by forced cocaine abstinence, and ADAR2 overexpression in the NAc shell attenuates cocaine-seeking behavior (Schmidt et al., 2015).

To investigate the roles of accumbal RNA editing in brain function, we produced NAc-specific conditional *ADAR2* knockout mice (*ADAR2*^flox/flox^; Hideyama et al., 2010), and performed anxiety- and depression-like behavioral tests, home-cage activity test, and ethanol drinking and ethanol conditioned place preference (CPP) tests.

## Materials and Methods

### Animals

All experiments were performed in accordance with international guidelines for the care and use of experimental animals. *ADAR2^flox/flox^* mice were maintained under a 12-h light-dark cycle (lights on from 08:00 to 20:00 h) with free access to water and food (Hideyama et al., 2010). Behavioral testing was performed between 9:00 and 18:00 h. After testing, the apparatus was cleaned with 70% ethanol to prevent bias caused by olfactory cues. All animal experiments, including production, maintenance protocols and behavioral studies, were reviewed and approved by the Animal Care and Use Committee of the Kyoto Prefectural University of Medicine (M26-250). Mice were anesthetized with a midazolam/medetomidine/butorphanol cocktail (4,0.3 and 5 mg/kg, respectively) during surgery. Prior to brain removal, all mice were euthanized with an excess of pentobarbital (50 mg/kg).

### Generation of a Conditional ADAR2 Knockout Mouse Using AAV-GFP/Cre

Male *ADAR2^flox/flox^* mice with a C57BL/6J genetic background (10–13 weeks old) were anesthetized and mounted in a stereotaxic apparatus (Narishige, Tokyo, Japan). AAV-GFP/Cre (Addgene plasmid # 49056) was a gift from Dr. Fred Gage (Kaspar et al., 2002). pAAV-GFP control vector was purchased from Cell Biolabs (San Diego, CA, USA). For recombinant AAV (rAAV) production, the vectors were cotransfected into HEK293 cells with pAAV-DJ and pHelper (Cell Biolabs), according to the manufacturer’s instructions. rAAV particles were purified by discontinuous step gradient using iodixanol (Opti-Prep; Nycomed Pharma, Oslo, Norway), as described previously (Aoki et al., 2016). Iodixanol fractions were concentrated and dialyzed by centrifugation through a Biomax 100K filter (Millipore, Billerica, MA, USA) with phosphate-buffered saline (PBS) containing 5% sorbitol. rAAV titers were determined by quantitative PCR and aliquoted rAAVs were stored at −80°C. AAV-GFP/Cre (1.63 × 10^13^ viral genome particles/ml) or AAV-GFP (2.2 × 10^12^ viral genome particles/mL) containing Fast Green dye (Nacalai Tesque, Kyoto, Japan) was bilaterally microinjected (1 μl/site) into the NAc (AP, +2 mm from Bregma; ML, ±0.8 mm from midline; DV, +4.3 mm below the skull surface) using a 30-gauge Hamilton syringe needle (Hamilton, Reno, NV, USA) at a rate of 0.2 μl/min. The needle was left in place for 12 min after each injection before slow retraction. GFP/Cre or GFP expression was allowed to develop for 4 weeks. After behavioral analysis, GFP/Cre and GFP gene expression were confirmed by immunohistochemical analysis using anti-GFP antibodies (MBL, Nagoya, Japan). Genomic DNA from the NAc was prepared using the NucleoSpin TriPrep kit (MACHEREY-NAGEL, Düren, Germany). Primers (5′-CTGGTTCATAACAGATCCTCAGGG-3′ and 5′-GTCTCCCTTGTCCTTCCAGGTAGC-3′) were used for genomic ADAR2 PCR (Hideyama et al., 2010).

### Immunohistochemical Analysis

Under deep anesthesia, AAV-GFP/Cre- or AAV-GFP- injected mice (*n* = 5) were perfused *via* the left cardiac ventricle with 20 ml chilled 0.1 M PBS followed by 40 ml of fixative containing 4% paraformaldehyde in 0.1 M phosphate buffer. Brains were post-fixed in the same fixative at 4°C overnight. After cryoprotection in 25% sucrose in 0.1 M PB for 48 h at 4°C, coronal sections (10-μm thick) were cut using a cryostat and mounted on coated glass slides (Fisherbrand Superfrost Plus; Fisher Scientific, Hampton, NH, USA). Sections were exposed to an antigen retrieval solution (Histo VT One; Nacalai Tesque, Kyoto, Japan) for 20 min at 70°C. After washing in PBS and incubating in blocking solution (Blocking One Histo; Nacalai Tesque, Kyoto, Japan), sections were incubated with mouse monoclonal anti-ADAR2 antibody (1:200; sc-73409, Santa Cruz Biotechnology Inc., Dallas, TX, USA) and rabbit monoclonal anti-GFP antibody (1:2,000; MBL, Nagoya, Japan) for 24 h at 4°C. For detection of primary antibodies, Alexa 594-conjugated goat anti-mouse and Alexa 488-conjugated goat anti-rabbit antibodies (1:500; Life Technologies, Carlsbad, CA, USA) were used. For NeuN detection, monoclonal anti-NeuN (1:200; MAB377, Merck, Burlington, MA, USA) and Alexa 594-conjugated goat anti-mouse (1:500; Life Technologies, Carlsbad, CA, USA) were used as primary and secondary antibodies, respectively. Sections were coverslipped and observed using an inverted fluorescence microscope (IX83 cellSense Dimension; Olympus, Tokyo, Japan) or inverted laser-scanning confocal microscope (LSM510 META; Carl Zeiss, Oberkochen, Germany). Images were captured as Z-stacks (8 z-sections, 0.5 μm apart).

### Measurement of RNA Editing Efficiency

*ADAR2^flox/flox^* mice were injected with AAV-GFP (*n* = 10) or AAV-GFP/Cre (*n* = 10) into the NAc. Mouse brains were obtained 1 month later, and quickly frozen in liquid nitrogen. Coronal sections (15 μm thick) were cut using a cryostat and mounted onto membrane slides (PEN-membrane 2.0 μm; Leica Microsystems, Wetzlar, Germany). NAc samples from mounted coronal sections were obtained by 1-mm punch biopsy, followed by RT-PCR amplification using VolcanoCell2G 2 × RT-PCR Master Mix (myPLOS Biotec, Konstanz, Germany). PCR products were treated with ExoSAP-IT (Affymetrix, Santa Clara, CA, USA) and subjected to sequencing reaction (BigDye Terminator v1.1 Cycle Sequencing Kit, ThermoFisher, Waltham, MA, USA). Primer sequences are described in Table 1. Sequencing analysis was performed with an Applied Biosystems 3130 Genetic Analyzer (ThermoFisher, Waltham, MA, USA). DNA sequencing data were converted to waveform data, and the peaks of adenosine (A) and guanosine (G) were quantified using ImageJ software^1^. RNA editing frequency was calculated as A/(A + G) × 100 (%).

**Table 1:** Primer sequences.

5-HT_2C_R Fw	5′-ATTGCTGATATGCTGGTGGGACTAC-3′
5-HT_2C_R Rv	5′-GCTTTCGTCCCTCAGTCCAATCACA-3′
CYFIP2 Fw	5′-AACTGGATGCCAAGAAGAGAATCAAC-3′
CYFIP2 Rv	5′-CAGCCCTGAGCCTGTCACCACCTCG-3′
GluR2 Fw	5′-AGCAGATTTAGCCCCTACGAG-3′
GluR2 Rv	5′-CAGCACTTTCGATGGGAGACAC-3′

### Behavioral Testing Experimental Design

*ADAR2^flox/flox^* mice were injected with AAV-GFP (*n* = 55) or AAV-GFP/Cre (*n* = 66) into the NAc. Mice were subjected to anxiety- and depression-like behavior tests and ethanol consumption tests. The order of each testing session was: (1) anhedonia test; (2) light/dark transition test; (3) open field test; (4) elevated plus maze (EPM); (5) forced swimming test; (6) tail suspension test (TST). After chronic ethanol exposure; (7) ethanol-drinking behavior test; and (8) CPP test were performed. A home-cage activity test was performed using an independent cohort of mice. The test battery was performed at 1–3 day inter-test intervals. We checked correct microinjection of AAV into the bilateral NAc after behavioral tests.

### Anhedonia Test

Mice were allowed free access to water and food prior to the anhedonia test. During testing, mice were given free choice between two bottles, one with water and another with 1% sucrose solution, for 24 h (Shirahase et al., 2016). Mice were allowed free access to food during the test period. After 12 h, the positions of the bottles were switched to exclude possible effects of side preference in drinking behavior. The consumption (ml) of sucrose solution and water were estimated after 24 h. Sucrose consumption was calculated as sucrose intake (g) per sucrose volume (ml) per body weight (kg). Water intake and total intake were calculated as the volume of fluid intake (ml) per body weight (kg).

### Light/Dark Transition Test

The light/dark transition test was performed as previously described (Aoki et al., 2016). The apparatus used for the light/dark transition test comprised a cage (14 × 14 cm) divided into two sections of equal size by a partition with a door (Melquest Co., Ltd, Toyama, Japan). One chamber was brightly illuminated (400 lux), whereas the other chamber was dark (2 lux). Mice were placed into the dark side and allowed to move freely between the two chambers with the door open for 10 min. The total number of transitions, time spent in each compartment, and latency of first movement to the light side were automatically recorded using an animal movement analysis system (SCANET-40; Melquest Co., Ltd).

### Open Field Test

Each subject was allowed to move freely in the open field box (60 × 60 cm) for 30 min. Time spent in the center area of the open field (30 × 30 cm) was measured using SMART v3.0 software (Panlab SL, Barcelona, Spain).

### Elevated Plus Maze (EPM)

The EPM test was performed as described previously (Aoki et al., 2016). The EPM consisted of two open arms (30 × 5 cm) and two enclosed arms of the same size, with 13-cm-high transparent walls. The arms and central square were constructed from gray plastic sheets elevated to a height of 50 cm above the floor. To minimize the likelihood of animals falling from the apparatus, 5-mm-high Plexiglas sides were used for the open arms. Arms of the same type were arranged at opposite sides to each other. The device was set up under low illumination (center square, 300 lux). Each mouse was placed in a closed arm of the maze. Mouse behavior was recorded during a 10-min test period. The number of entries into and the time spent on open arms were recorded. For data analysis, the following two measures were used: percentage of entries into open arms and duration of stay on open arms. Data acquisition and analysis were performed using SMART v3.0 software.

### Forced Swim Test (FST)

The FST apparatus consisted of four Plexiglas cylinders. The cylinders were filled with water at a temperature of 23°C up to a height of 10 cm. Mice were placed into the cylinders and images recorded over a 10-min period. Data acquisition and analysis were performed using SMART v3.0 software. Immobility time was measured during 2–6 min of the test period (Aoki et al., 2016).

### Tail Suspension Test (TST)

The TST was performed according to a previously described procedure (Watanabe et al., 2011). Mice were suspended 30 cm above the floor in a visually isolated area using adhesive tape placed 1 cm from the tip of the tail. Immobility duration was recorded over a 10-min test period. Images were captured using a USB camera (Webcam C270; Logicool, Tokyo, Japan). Data acquisition and analysis were performed using SMART v3.0 software.

### Home-Cage Activity Test

Mice were placed in a home cage (22 × 22 × 18.5 cm) for 7 days prior to the home-cage activity test. Home-cage activity was recoded for 3 days using an animal movement analysis system (SCANET-40). Mice were maintained under a 12-h light/dark cycle (lights on from 08:00 to 20:00 h) with free access to water and food.

### Chronic Ethanol Vapor Exposure

Chronically ethanol-exposed mice were produced as previously described (Yoshimoto et al., 2012). Briefly, mice were exposed to 22–27 mg/l of ethanol vapor during dark phase for 20 days using an intermittent 3–6 h/day schedule that mimics cyclical patterns of ethanol consumption. Blood ethanol concentration of mice was not measured. Mice were maintained in the ethanol vapor chamber under a 12-h light/dark cycle (lights on from 08:00 to 20:00 h) with free access to water and food.

### Ethanol-Drinking Behavior Test

After chronic ethanol exposure, mice were withdrawn from ethanol and maintained under normal experimental conditions for 4 h. For the ethanol-drinking behavior test, mice were provided with 10% (v/v) ethanol solution for 4 h, and their consumption was measured (Watanabe et al., 2014). The total amount of ethanol intake was represented as ethanol (g)/body weight (kg).

### Ethanol CPP Test

The CPP apparatus was divided into two sections of equal size (14 × 14 cm) by a partition with a door (Melquest Co. Ltd, Toyama, Japan). One box was painted black with a smooth floor and the other was painted white with a textured floor. The CPP test was performed 1–2 days after the ethanol drinking behavior test according to a previously described procedure (Thanos et al., 2005). All mice were allowed to recover under normal conditions for 3–5 days before the conditioning phase. In the preconditioning phase (days 1–3), each mouse was placed in the CPP apparatus for 30 min. Data from the 3rd day was analyzed for any unconditioned box preference. In the conditioning phase (days 4–11), mice were given saline in the black box on days 4, 6, 8, and 10. On days 5, 7, 9, and 11, the same mice were given ethanol (2 g/kg i.p. 20% (v/v) EtOH in saline) in the opposite chamber (for 30 min/trial). In the test phase (day 12), mice were placed in the apparatus and allowed free access to all boxes for 30 min. Each test session (days 3 and 12) was automatically recorded using an animal movement analysis system (SCANET-40).

### Statistical Analysis

Statistical analysis was performed using Stat View (SAS Institute, Cary, NC, USA). Significance of differences between two groups was calculated using Student’s *t*-tests. Ethanol-drinking behavior and CPP test data were analyzed by two-way analyses of variance (ANOVA; Bonferroni’s *post hoc* test). The alpha level was set to 0.05. Results of statistical analysis of RNA editing efficiency and behavioral tests were summarized in Table 2.

**Table 2:** Summary of statistical analysis.

**Tests and factors**	*F* and *p* values	Statistical analysis
**Ratio of RNA editing**		
5-HT_2C_R Site D NAc	*p = 0.0006	*t*-test
5-HT_2C_R Site D Cortex	*p* = 0.8137	*t*-test
CYFIP2 K/E NAc	*p = 0.00000006	*t*-test
CYFIP2 K/E Cortex	*p* = 0.6301	*t*-test
GluA2 Q/R NAc	*p = 0.00001	*t*-test
GluA2 Q/R Cortex	*p* = 0.4893	*t*-test
**Body weight**	*p* = 0.7417	*t*-test
**Light/Dark test**		
Transitions	*p = 0.0069	*t*-test
Stay time in light	*p* = 0.1986	*t*-test
Latency to light	*p* = 0.131	*t*-test
**Open field test**		
Center time (s)	*p* = 0.3882	*t*-test
Center time (%)	*p* = 0.4949	*t*-test
Total distance	*p = 0.0058	*t*-test
**Elevated plus maze**		
Number of entries	*p* = 0.2828	*t*-test
Entries into open arms	*p* = 0.9678	*t*-test
Time on open arms	*p* = 0.7682	*t*-test
Total distance	*p* = 0.5778	*t*-test
**Spontaneous locomotor activity**		
Light	*p* = 0.6986	*t*-test
Dark	*p* = 0.1748	*t*-test
**Forced swim test**	*p* = 0.1571	*t*-test
**Tail suspension test**	*p* = 0.2692	*t*-test
**Sucrose test**		
1% Sucrose consumption	*p* = 0.2236	*t*-test
Water	*p* = 0.9768	*t*-test
Total intake	*p* = 0.2113	*t*-test
**Ethanol drinking test**		
Ethanol vapor	*F*_(1,35)_ = 6.687, *p* = 0.14	ANOVA
Genotype	*F*_(1,35)_ = 0.725, *p* = 0.4004	ANOVA
Ethanol vapor × Genotype	*F*_(1,35)_ = 4.911, **p = 0.0333	ANOVA
*Post hoc* (Control vs. Ethanol vapor)		
GFP	*p = 0.0009	Bonferroni
GFP/Cre	*p* = 0.8068	Bonferroni
**CPP test (Control)**		
Conditioning	*F*_(1,40)_ = 0.734, *p* = 0.3968	ANOVA
Genotype	*F*_(1,40)_ = 0.11, *p* = 0.7423	ANOVA
Conditioning × Genotype	*F*_(1,40)_ = 0.18, *p* = 0.6736	ANOVA
*Post hoc* (Control vs. Ethanol vapor)		
GFP	*p* = 0.3478	Bonferroni
GFP/Cre	*p* = 0.7713	Bonferroni
**CPP test (Ethanol vapor)**		
Conditioning	*F*_(1,34)_ = 7.194, **p = 0.0112	ANOVA
Genotype	*F*_(1,34)_ = 0.042, *p* = 0.8392	ANOVA
Conditioning × Genotype	*F*_(1,34)_ = 0.63, *p* = 0.4328	ANOVA
*Post hoc* (Control vs. Ethanol vapor)		
GFP	**p = 0.0222	Bonferroni
GFP/Cre	*p* = 0.1791	Bonferroni

*p < 0.01, **p < 0.05.

## Results

### Conditional Knockout of ADAR2 in the NAc Using AAV-GFP/Cre

**Figure 1:**
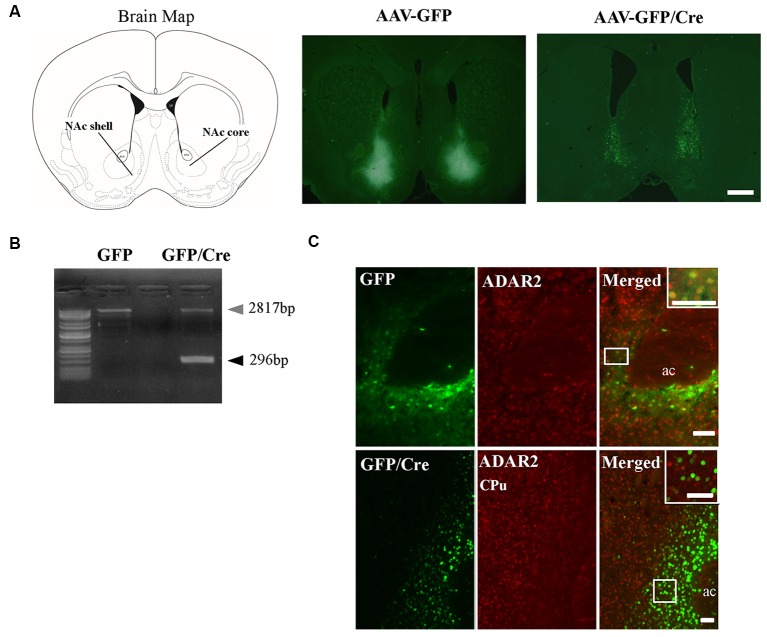
Nucleus accumbens (NAc)-specific *ADAR2* knockout mice. **(A)** AAV-GFP or AAV-GFP/Cre was stereotaxically injected into the NAc of *ADAR2*^flox/flox^ mice, based on the coordinates of Paxinos and Franklin (Paxinos and Franklin, 2004), and NAc sections were examined for GFP or GFP/Cre expression using anti-GFP antibody. Scale bar: 500 μm. **(B)** Knockout of the double-stranded RNA-specific adenosine deaminase *ADAR2* was validated by a genomic PCR assay. *ADAR2* was deleted by Cre-lox recombination (296 bp). **(C)** Ten micrometer coronal brain sections from *ADAR2*^flox/flox^ mice injected with AAV-GFP (upper) or AAV-GFP/Cre (lower) into the NAc were immunohistochemically examined using anti-GFP (green) and anti-ADAR2 (red) antibodies. ac, anterior commissure; CPu, caudate putamen. Scale bars: 50 μm.

**Figure 2:**
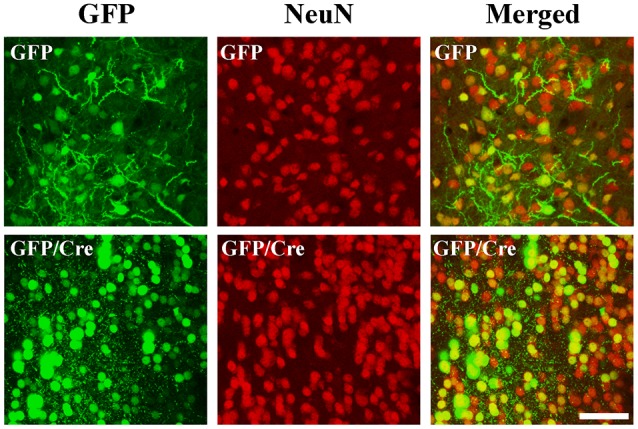
Survival of ADAR2-knockout neurons in the NAc. AAV-GFP or AAV-GFP/Cre was stereotaxically injected into the NAc of *ADAR2^flox/flox^* mice. Coronal brain sections in the NAc were immunohistochemically examined using anti-GFP (green) and anti-NeuN (red) antibodies. Scale bars: 50 μm.

**Figure 3:**
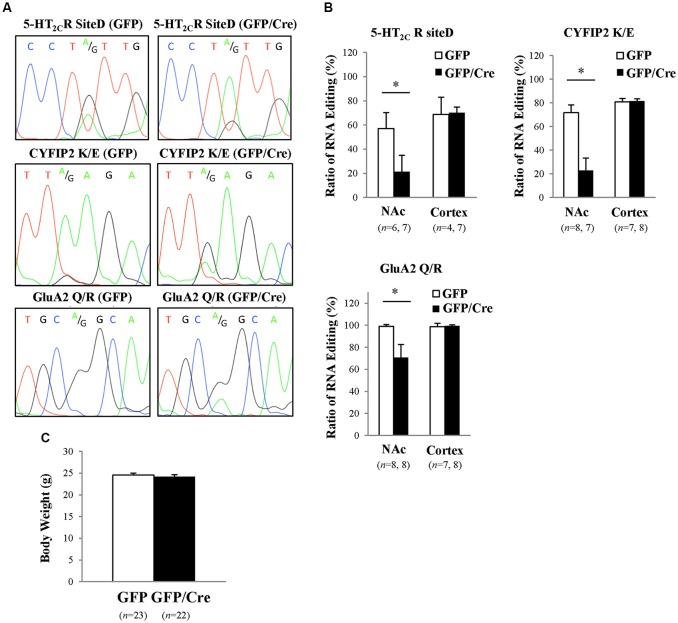
Reduced RNA editing in the NAc of *ADAR2* knockout mice. **(A)** Suppression of ADAR2-mediated RNA editing; 5-HT_2C_R site D (upper), CYFIP2 K/E site (middle), and GluA2 Q/R site (lower). Total RNA was prepared from the NAc of AAV-GFP- or AAV-GFP/Cre-injected mice. cDNAs were synthesized from total RNA, followed by direct sequencing analysis. Editing sites were indicated by A/G in waveform data. **(B)** Editing frequency was calculated based on sequence trace data. Samples were prepared from the NAc and prefrontal cortex of AAV-GFP- (open squares) or AAV-GFP/Cre- (closed squares) injected mice. *p < 0.01. **(C)** Body weights of AAV-GFP- or AAV-GFP/Cre-injected mice 4 weeks after AAV injection.

To generate NAc-specific *ADAR2* knockout mice with a C57BL/6J genetic background, AAV-GFP/Cre was injected into the NAc (core and shell) of *ADAR2*^flox/flox^ mice (Figure 1A). Injection into the NAc showed about 500 μm diameter spread of AAV. Genomic PCR analysis showed that the recombination of *ADAR2*^flox/flox^ gene by Cre recombinase occurred in the NAc of AAV-GFP/Cre-injected mice (Figure 1B). In immunohistochemical analysis, ADAR2 expression was not detected in AAV-infected GFP/Cre-positive neurons in the NAc, whereas both ADAR2 and GFP expression were observed in the control AAV-GFP-infected neurons (Figure 1C). The loss of ADAR2 expression in AAV-GFP/Cre-injected NAc was not due to cell death, because numbers of NeuN-positive cells were not reduced and both NeuN- and GFP-positive neurons were observed in control AAV-GFP-injected NAc (Figure 2). Four weeks after the injection, we examined the influence of *ADAR2*-knockout on RNA editing. Sections from the NAc were lysed, and cDNAs were synthesized. The RNA editing frequency of ADAR2-dependent RNA editing sites, GluA2 Q/R, 5-HT_2C_R site D and CYFIP2 K/E, was measured by direct sequencing analysis of cDNAs. Compared with that of AAV-GFP-injected mice, the RNA editing frequency of the three editing sites was significantly lower in the NAc of NAc-specific *ADAR2* knockout mice (Figures 3A,B). The editing frequency of the ADAR2-mediated RNA editing sites 5-HT_2C_R site D and CYFIP2 K/E site was reduced from 60% to 70% in controls to approximately 20% in *ADAR2* knockout mice. Editing frequency of the GluA2 Q/R site was also lower in *ADAR2* knockout mice (60%, compared with 100% in control mice). In contrast, the RNA editing frequency of all sites in the cortex was normal (Figures 3A,B). The body weight of control (GFP) and NAc-specific *ADAR2* knockout mice (Cre) was measured 4 weeks after AAV injection. All mice appeared to be healthy, and mean body weight was not significantly different between the groups (Figure 3C).

### Analysis of Anxiety-Related Behaviors of NAc-Specific ADAR2 Knockout Mice

**Figure 4:**
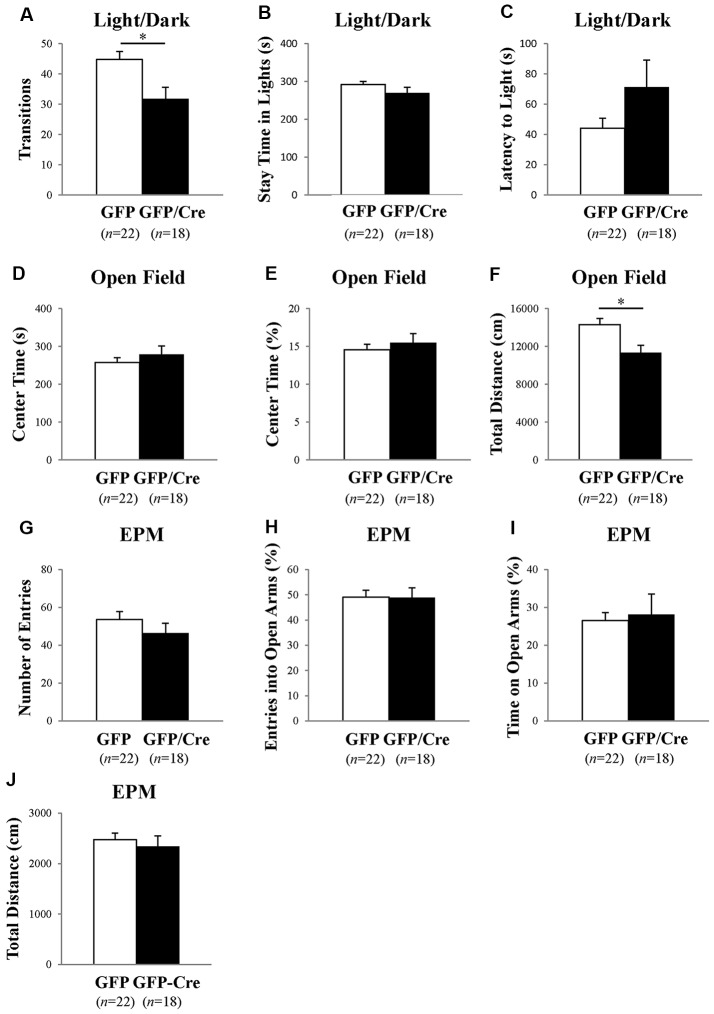
Analysis of anxiety-like behaviors and locomotion in NAc-specific *ADAR2* knockout mice. AAV-GFP- (open squares) or AAV-GFP/Cre- (closed squares) injected mice were subjected to light/dark test **(A–C)**, open field test **(D,E)**, and elevated plus maze (EPM) test** (G–J)**. In the light/dark test, the number of light/dark transitions **(A)**, time spent in light compartment **(B)** and latency to enter the light compartment **(C)** were recorded. In the open field test, time spent in the center of the compartment **(D)** and total locomotor distance traveled **(E)** were recorded. In the EPM test, the number of arm entries **(F)**, percentage of entry into open arms **(G)**, time spent in open arms **(H)** and distance traveled **(I)** were recorded. Sample numbers are indicated in parentheses. Data are presented as mean ± SEM. *P* values were calculated using Student’s *t*-test. *p < 0.01.

**Figure 5:**
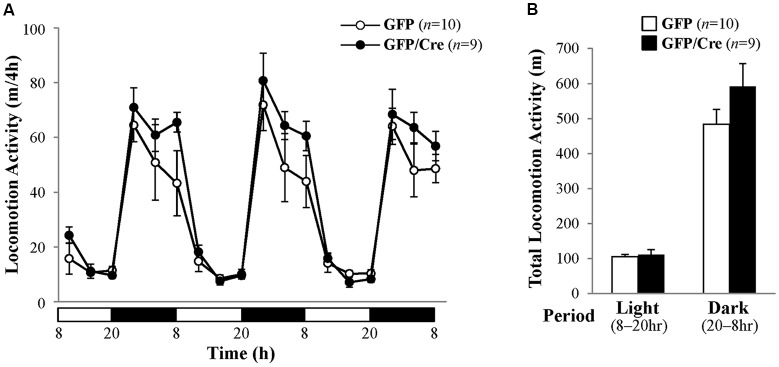
Analysis of spontaneous locomotor activity. **(A)** Spontaneous locomotor activity of AAV-GFP- (*n* = 10) or AAV-GFP/Cre- (*n* = 9) injected mice was recorded for 3 days under 12 h light/dark cycle after familiarization to the new cage environment. Each point represents the 4-h average of locomotor activity. The day was divided into a 12-h light period (white bars) and a 12-h dark period (black bars). **(B)** Total locomotor activity for 3 days is presented as mean ± SEM. *P* values were calculated using Student’s *t*-test.

We characterized the behavioral phenotypes of NAc-specific *ADAR2* knockout mice 4 weeks after the AAV injection. Mice were subjected to a light/dark transition test, open field test and EPM test as analyses of anxiety-related behaviors. In the light/dark transition test, the total number of transitions between the light and dark chambers was significantly lower in NAc-specific *ADAR2* knockout mice (*p* = 0.0069; Figure 4A), while there were no significant differences in the stay time in the light chamber or latency to first enter the light chamber (Figures 4B,C). In the open field test, no significant difference between the two groups was observed in the time spent in the central area (Figures 4D,E). However, the distance traveled by NAc-specific *ADAR2* knockout mice was significantly lower than that of control mice (*p* = 0.0058; Figure 4F). In the EPM test, major differences were not seen in number and percentage of entries into the open arms, percentage of stay time in the open arm or the distance traveled (Figures 4G–J). A diurnal rhythm of spontaneous locomotor activity was recorded for 3 days. Both NAc-specific *ADAR2* knockout mice and control mice showed normal rhythms and similar gross levels of spontaneous locomotor activity (Figure 5).

### Analysis of Despair- and Anhedonia-Related Behaviors of NAc-Specific ADAR2 Knockout Mice

**Figure 6:**
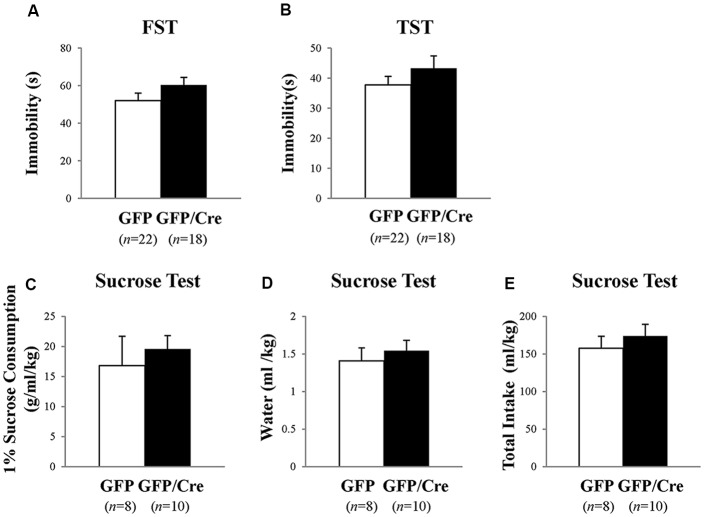
Analysis of despair-like behaviors and anhedonia test. AAV-GFP- (open squares) or AAV-GFP/Cre- (closed squares) injected mice were subjected to forced swimming test (FST; **A**), tail suspension test (TST; **B**) and anhedonia test **(C–E)**. Immobility time was recorded **(A,B)**. Sucrose preference was measured as an indicator of anhedonia. Sucrose consumption **(C)**, water consumption **(D)** and total intake **(E)** were recorded for 24 h. Sample numbers are indicated in parentheses. Data are presented as mean ± SEM. *P* values were calculated using Student’s *t*-test.

Next, we examined despair-related behaviors using the FST and TST. The results of the FST revealed no significant difference in immobility time between control and NAc-specific *ADAR2* knockout mice (Figure 6A). Similarly, there was no significant difference in immobility time in the TST (Figure 6B). Mice were subjected to the sucrose preference test as a measure of anhedonia-related behavior. Mice had the choice of either drinking 1% sucrose solution or water for 24 h. There were no differences in the consumption of sucrose solution or water between the two groups (Figures 6C–E). These results showed that despair- and anhedonia-related phenotypes of NAc-specific *ADAR2* knockout mice were normal.

### Ethanol-Drinking Behavior and Ethanol CPP Tests

**Figure 7:**
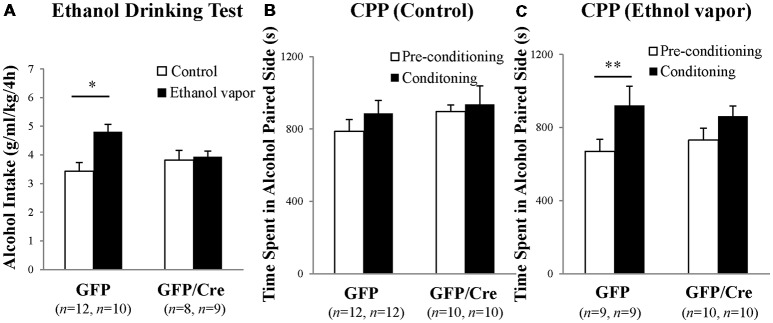
Ethanol-drinking behavior and ethanol preference. AAV-GFP- (open squares) or AAV-GFP/Cre- (closed squares) injected mice were exposed to ambient air (Control) or ethanol vapor (EtOH) for 20 days. Ten percentage ethanol consumption within a 4-h period was recorded **(A)**. Data are presented as mean ± SEM. Statistical analyses were performed using two-way analyses of variance (ANOVA), followed by Bonferroni’s *post hoc* test. Subsequently, these mice were subjected to the ethanol-induced conditioned place preference (CPP) test 1–2 days after the ethanol drinking test **(B,C)**. Time spent in the ethanol-paired side was measured in the pre-conditioning (open squares) and post-conditioning (closed squares) phase. Sample numbers are indicated in parentheses. Data are presented as mean ± SEM. Statistical analyses were performed using two-way ANOVA, followed by Bonferroni’s *post hoc* test. *p < 0.01 and **p < 0.05.

GFP and Cre/GFP mice were exposed to ethanol vapor for 20 days prior to ethanol-drinking behavior and ethanol preference tests. Chronic ethanol vapor exposure significantly increased 10% ethanol intake in GFP mice (genotype × treatment: *F*_(1,35)_ = 4.911, *p* = 0.0333, two-way ANOVA; GFP-control vs. GFP-ethanol vapor: *p* = 0.0009, Bonferroni’s *post hoc* test), while Cre/GFP mice did not show enhanced ethanol intake (Figure 7A). This result suggests that ADAR2-mediated RNA editing in the NAc is involved in ethanol-drinking behavior. Following the ethanol-drinking behavior test, ethanol preference was measured in these mice using the ethanol CPP test. In control mice, there was no significant difference in CPP score between GFP and Cre/GFP mice, and the mice in both cases did not develop ethanol CPP (Figure 7B). In contrast, there was a significant difference in the CPP scores of GFP mice after chronic ethanol exposure between the pre-conditioning and conditioning phases (Figure 7C; ethanol-conditioned: *F*_(1,34)_ = 7.194, *p* = 0.0112, two-way ANOVA; *p* = 0.0222 *post hoc* test), whereas there was no significant difference in the CPP scores of Cre/GFP mice after chronic ethanol exposure between the pre-conditioning and conditioning phases (Figure 7C). These results indicate that ADAR2-mediated RNA editing in the NAc is involved in the enhancement of ethanol preference induced by chronic ethanol exposure.

## Discussion

In the present study, we demonstrated that ADAR2-mediated RNA editing in the NAc underlies enhanced ethanol consumption and preference after chronic ethanol exposure. ADAR2 mediates RNA editing of various ion channels and receptors such as Ca_V_1.3 calcium ion channel, K_V_1.1 potassium ion channel, 5-HT_2C_R, GluA2, and GABA_A_ (Bhalla et al., 2004; Bazzazi et al., 2013; Behm and Öhman, 2016). We previously reported that 5-HT_2C_R RNA editing is involved in voluntary alcohol consumption after chronic ethanol vapor exposure (Watanabe et al., 2014). In C57BL/6J mice, chronic ethanol vapor exposure causes elevated 5-HT_2C_R RNA editing in the NAc and DRN by upregulation of ADAR1 and ADAR2 expression. On the other hand, INI mice, in which 5-HT_2C_R RNA editing was blocked by the deletion of the editing complementary sequence in intron 5 (160 bp), showed no enhanced ethanol consumption (Watanabe et al., 2014). In light of previous findings, our results may imply that a reduction in 5-HT_2C_R RNA editing causes suppression of enhanced ethanol consumption and ethanol preference after chronic ethanol vapor exposure in NAc-specific *ADAR2* knockout mice. Besides 5-HT_2C_R RNA editing, RNA editing of other receptors and ion channels may be also involved in alcohol-related behaviors. For example, activation of GABA_A_ receptor by RNA editing may enhance ethanol consumption and preference. An increase in GABAergic terminal density was found in the NAc of ethanol-preferring rats and high-ethanol-drinking rats (Hwang et al., 1990). The RNA-edited isoform of the GABA_A_ α3 subunit exhibits greater GABA sensitivity and slower decay (Nimmich et al., 2009), suggesting that a reduction in GABA_A_ α3 RNA editing may also cause suppression of enhanced ethanol consumption and ethanol preference in NAc-specific *ADAR2* knockout mice. Moreover, GluA2 RNA editing in the NAc may play a role in ethanol drinking behavior and ethanol preference. ADAR2-mediated RNA editing in the NAc is also involved in cocaine-seeking behavior. In contrast to ethanol preference, however, ADAR2-mediated RNA editing negatively affects cocaine seeking. Cocaine abstinence decreases ADAR2 expression in the NAc, resulting in reduction of GluA2 Q/R site editing, and unedited GluA2 promotes cocaine seeking (Schmidt et al., 2015). Furthermore, overexpression of ADAR2 in the NAc shell attenuates cocaine priming-induced reinstatement (Schmidt et al., 2015). The apparent discrepancy between the involvement of RNA editing in different types of addiction might be attributed to the distinct neuronal circuits and RNA-edited receptors underlying addiction to alcohol and cocaine. Collectively, the phenotype of accumbal *ADAR2* knockout mice in ethanol drinking and preference may be the total outcome of changes in multiple genes influenced by RNA editing, including 5-HT_2C_R.

Neuropharmacological studies have shown that multiple 5-HT receptor subtypes, such as 5-HT_1A/1B_R, 5-HT_2A/2C_R, 5-HT_3_R, and 5-HT_7_R, are involved in the development of alcohol dependence (Sari et al., 2011; Yoshimoto et al., 2012; Hauser et al., 2015). Serotonergic system draws attention as a therapeutic target for alcohol dependence. Previously, we reported that wild type mice with a C57BL/6J genetic background predominantly express edited VXV isoforms of 5-HT_2C_R (59%) in the NAc, including VNV, VGV and VSV isoforms (Watanabe et al., 2014). In the present study, the frequency of 5-HT_2C_R RNA editing at site D was reduced to 20% in the NAc of *ADAR2* knockout mice (Figure 3B), meaning there was a reduction of expression of VXV isoforms. Compared with 5-HT_2C_R VXV isoforms, other isoforms such as VNI and VGI, which are generated by blocking ADAR2-mediated RNA editing, have higher receptor activity (Burns et al., 1997; Niswender et al., 1999; Watanabe et al., 2014). 5-HT_2C_R activity negatively modulates mesoaccumbens dopamine neurons through inhibition of dopamine release (De Deurwaerdère et al., 2004; Di Matteo et al., 2004). Dopaminergic dysfunction in the mesoaccumbens caused by long-term alcohol intake is known to be involved in alcoholism (Heinz, 2002). Thus, a reduction of accumbal dopamine signaling caused by enhanced RNA editing of 5-HT_2C_R might contribute to an increase in ethanol drinking and preference after chronic ethanol exposure. Indeed, alcohol consumption influences levels of neurotransmitters, such as dopamine and 5-HT (Yoshimoto et al., 1996; Tattoli et al., 2001). The release of them in the NAc is altered during chronic consumption and withdrawal of alcohol, underlying the development of alcohol use disorder (Banerjee, 2014). Thus, the dopamine and 5-HT pathways might be influenced by the alteration of 5-HT_2C_R RNA editing by chronic ethanol exposure, resulting in enhanced alcohol consumption.

It is thought that RNA editing is involved in many psychiatric disorders, including mood disorder and major depression (Tariq and Jantsch, 2012; Weissmann et al., 2016). Heterozygous *ADAR2* knockout mice showed a tendency of resistance to behavioral despair (Kubota-Sakashita et al., 2014). While we have previously reported that INI mice show a despair phenotype associated with accumbal NPY expression (Mombereau et al., 2010; Aoki et al., 2016), in this study, we did not observe this phenotype in NAc-specific *ADAR2* knockout mice (Figure 6A). A possible explanation for this difference is that the despair phenotype might be masked by the reduction of RNA editing of other gene transcripts such as AMPA receptors. Alternatively, further reduction of 5-HT_2C_R RNA editing might be required to display severe phenotype since other editing sites of 5-HT_2C_R which are mediated by ADAR1 is normal in NAc-specific *ADAR2* knockout mice.

In the present study, there were no significant difference in CPP scores between pre-conditioning and conditioning of control mice injected with GFP (Figure 7B). This might be due to the influence of the ethanol drinking test prior to the CPP test. All mice used in the CPP test had an experience of ethanol drinking for a short time. In further study, the will be performed using an independent cohort of mice.

In conclusion, in the present study, using NAc-specific *ADAR2* knockout mice, we have demonstrated that RNA editing in the NAc is involved in ethanol preference. Further study of the relationship between RNA editing and alcohol use disorder in humans will provide more insights, and could lead to a novel treatment for alcohol use disorder in the future.

## Author Contributions

MT and YW designed the experiments. TS and YW performed behavior tests, sequencing analysis, and statistical analysis. AT performed AAV production. MT and TS performed immunohistochemistry. SK provided advice for transgenic mice and sequencing analysis. TS, YW, and MT wrote the manuscript. TS, YW, SK, TY, NK, and MT discussed the results and commented on the manuscript.

## Conflict of Interest Statement

The authors declare that the research was conducted in the absence of any commercial or financial relationships that could be construed as a potential conflict of interest.

## Footnotes

^1^http://rsb.info.nih.gov/ij

## References

[B1] Aoki M. Watanabe Y. Yoshimoto K. Tsujimura A. Yamamoto T. Kanamura N.. (2016). Involvement of serotonin 2C receptor RNA editing in accumbal neuropeptide Y expression and behavioural despair. Eur. J. Neurosci. 43, 1219–1228. 10.1111/ejn.13233 26950265

[B2] Banerjee N. (2014). Neurotransmitters in alcoholism: a review of neurobiological and genetic studies. Indian J. Hum. Genet. 20, 20–31. 10.4103/0971-6866.132750 24959010 PMC4065474

[B3] Bazzazi H. Ben Johny M. Adams P. J. Soong T. W. Yue D. T. (2013). Continuously tunable Ca(2+) regulation of RNA-edited CaV1.3 channels. Cell Rep. 5, 367–377. 10.1016/j.celrep.2013.09.006 24120865 PMC4349392

[B4] Behm M. Öhman M. (2016). RNA editing: a contributor to neuronal dynamics in the mammalian brain. Trends Genet. 32, 165–175. 10.1016/j.tig.2015.12.005 26803450

[B5] Bhalla T. Rosenthal J. J. Holmgren M. Reenan R. (2004). Control of human potassium channel inactivation by editing of a small mRNA hairpin. Nat. Struct. Mol. Biol. 11, 950–956. 10.1038/nsmb825 15361858

[B6] Burns C. M. Chu H. Rueter S. M. Hutchinson L. K. Canton H. Sanders-Bush E.. (1997). Regulation of serotonin-2C receptor G-protein coupling by RNA editing. Nature 387, 303–308. 10.1038/387303a0 9153397

[B7] De Deurwaerdère P. Navailles S. Berg K. A. Clarke W. P. Spampinato U. (2004). Constitutive activity of the serotonin2C receptor inhibits *in vivo* dopamine release in the rat striatum and nucleus accumbens. J. Neurosci. 24, 3235–3241. 10.1523/JNEUROSCI.0112-04.2004 15056702 PMC6730027

[B8] Di Matteo V. Pierucci M. Esposito E. (2004). Selective stimulation of serotonin2c receptors blocks the enhancement of striatal and accumbal dopamine release induced by nicotine administration. J. Neurochem. 89, 418–429. 10.1111/j.1471-4159.2004.02337.x 15056285

[B9] Fitzgerald L. W. Iyer G. Conklin D. S. Krause C. M. Marshall A. Patterson J. P.. (1999). Messenger RNA editing of the human serotonin 5-HT_2C_ receptor. Neuropsychopharmacology 21, 82S–90S. 10.1016/S0893-133X(99)00004-4 10432493

[B10] Gurevich I. Englander M. T. Adlersberg M. Siegal N. B. Schmauss C. (2002). Modulation of serotonin 2C receptor editing by sustained changes in serotonergic neurotransmission. J. Neurosci. 22, 10529–10532. 10.1523/jneurosci.22-24-10529.2002 12486144 PMC6758441

[B11] Hauser S. R. Hedlund P. B. Roberts A. J. Sari Y. Bell R. L. Engleman E. A. (2015). The 5-HT7 receptor as a potential target for treating drug and alcohol abuse. Front. Neurosci. 8:448. 10.3389/fnins.2014.00448 25628528 PMC4292232

[B12] Heinz A. (2002). Dopaminergic dysfunction in alcoholism and schizophrenia—psychopathological and behavioral correlates. Eur. Psychiatry 17, 9–16. 10.1016/S0924-9338(02)00628-4 11918987

[B13] Hideyama T. Yamashita T. Suzuki T. Tsuji S. Higuchi M. Seeburg P. H.. (2010). Induced loss of ADAR2 engenders slow death of motor neurons from Q/R site-unedited GluR2. J. Neurosci. 30, 11917–11925. 10.1523/JNEUROSCI.2021-10.2010 20826656 PMC6633551

[B14] Hwang B. H. Lumeng L. Wu J. Y. Li T. K. (1990). Increased number of GABAergic terminals in the nucleus accumbens is associated with alcohol preference in rats. Alcohol. Clin. Exp. Res. 14, 503–507. 10.1111/j.1530-0277.1990.tb01188.x 2171372

[B15] Kaspar B. K. Vissel B. Bengoechea T. Crone S. Randolph-Moore L. Muller R.. (2002). Adeno-associated virus effectively mediates conditional gene modification in the brain. Proc. Natl. Acad. Sci. U S A 99, 2320–2325. 10.1073/pnas.042678699 11842206 PMC122363

[B16] Kawahara Y. Grimberg A. Teegarden S. Mombereau C. Liu S. Bale T. L.. (2008). Dysregulated editing of serotonin 2C receptor mRNAs results in energy dissipation and loss of fat mass. J. Neurosci. 28, 12834–12844. 10.1523/JNEUROSCI.3896-08.2008 19036977 PMC2615198

[B17] Kawahara Y. Ito K. Sun H. Aizawa H. Kanazawa I. Kwak S. (2004). Glutamate receptors: RNA editing and death of motor neurons. Nature 427:801. 10.1038/427801a 14985749

[B18] Kubota-Sakashita M. Iwamoto K. Bundo M. Kato T. (2014). A role of ADAR2 and RNA editing of glutamate receptors in mood disorders and schizophrenia. Mol. Brain 7:5. 10.1186/1756-6606-7-5 24443933 PMC3902024

[B19] Martin C. B. Ramond F. Farrington D. T. Aguiar A. S. Jr. Chevarin C. Berthiau A. S.. (2013). RNA splicing and editing modulation of 5-HT_2C_ receptor function: relevance to anxiety and aggression in VGV mice. Mol. Psychiatry 18, 656–665. 10.1038/mp.2012.171 23247076

[B20] Mombereau C. Kawahara Y. Gundersen B. B. Nishikura K. Blendy J. A. (2010). Functional relevance of serotonin 2C receptor mRNA editing in antidepressant- and anxiety-like behaviors. Neuropharmacology 59, 468–473. 10.1016/j.neuropharm.2010.06.009 20624407 PMC2946438

[B21] Nimmich M. L. Heidelberg L. S. Fisher J. L. (2009). RNA editing of the GABA_A_ receptor α3 subunit alters the functional properties of recombinant receptors. Neurosci. Res. 63, 288–293. 10.1016/j.neures.2009.01.003 19367790 PMC2775542

[B22] Nishikura K. (2016). A-to-I editing of coding and non-coding RNAs by ADARs. Nat. Rev. Mol. Cell Biol. 17, 83–96. 10.1038/nrm.2015.4 26648264 PMC4824625

[B23] Niswender C. M. Copeland S. C. Herrick-Davis K. Emeson R. B. Sanders-Bush E. (1999). RNA editing of the human serotonin 5-hydroxytryptamine 2C receptor silences constitutive activity. J. Biol. Chem. 274, 9472–9478. 10.1074/jbc.274.14.9472 10092629

[B24] Niswender C. M. Sanders-Bush E. Emeson R. B. (1998). Identification and characterization of RNA editing events within the 5-HT_2C_ receptor. Ann. N Y Acad. Sci. 861, 38–48. 10.1111/j.1749-6632.1998.tb10171.x 9928237

[B25] Paxinos G. Franklin K. B. J. (2004). The Mouse Brain in Stereotaxic Coordinates, Second Edition. New York, NY: Elsevier.

[B26] Sari Y. Johnson V. R. Weedman J. M. (2011). Role of the serotonergic system in alcohol dependence: from animal models to clinics. Prog. Mol. Biol. Transl. Sci. 98, 401–443. 10.1016/B978-0-12-385506-0.00010-7 21199778 PMC3508458

[B27] Schmidt H. D. McFarland K. N. Darnell S. B. Huizenga M. N. Sangrey G. R. Cha J. H.. (2015). ADAR2-dependent GluA2 editing regulates cocaine seeking. Mol. Psychiatry 20, 1460–1466. 10.1038/mp.2014.134 25349168 PMC4412769

[B28] Shirahase T. Aoki M. Watanabe R. Watanabe Y. Tanaka M. (2016). Increased alcohol consumption in relaxin-3 deficient male mice. Neurosci. Lett. 612, 155–160. 10.1016/j.neulet.2015.12.014 26687275

[B29] Sommer B. Köhler M. Sprengel R. Seeburg P. H. (1991). RNA editing in brain controls a determinant of ion flow in glutamate-gated channels. Cell 67, 11–19. 10.1016/0092-8674(91)90568-j 1717158

[B30] Tariq A. Jantsch M. F. (2012). Transcript diversification in the nervous system: a to I RNA editing in CNS function and disease development. Front. Neurosci. 6:99. 10.3389/fnins.2012.00099 22787438 PMC3391646

[B31] Tattoli M. Cagiano R. Gaetani S. Ghiglieri V. Giustino A. Mereu G.. (2001). Neurofunctional effects of developmental alcohol exposure in alcohol-preferring and alcohol-nonpreferring rats. Neuropsychopharmacology 24, 691–705. 10.1016/S0893-133X(00)00225-6 11331149

[B32] Thanos P. K. Dimitrakakis E. S. Rice O. Gifford A. Volkow N. D. (2005). Ethanol self-administration and ethanol conditioned place preference are reduced in mice lacking cannabinoid CB1 receptors. Behav. Brain Res. 164, 206–213. 10.1016/j.bbr.2005.06.021 16140402

[B33] Wang Q. O’Brien P. J. Chen C. X. Cho D. S. Murray J. M. Nishikura K. (2000). Altered G protein-coupling functions of RNA editing isoform and splicing variant serotonin2C receptors. J. Neurochem. 74, 1290–1300. 10.1046/j.1471-4159.2000.741290.x 10693963

[B34] Watanabe Y. Tsujimura A. Takao K. Nishi K. Ito Y. Yasuhara Y.. (2011). Relaxin-3-deficient mice showed slight alteration in anxiety-related behavior. Front. Behav. Neurosci. 5:50. 10.3389/fnbeh.2011.00050 21887138 PMC3156976

[B35] Watanabe Y. Yoshimoto K. Tatebe H. Kita M. Nishikura K. Kimura M.. (2014). Enhancement of alcohol drinking in mice depends on alterations in RNA editing of serotonin 2C receptors. Int. J. Neuropsychopharmacol. 17, 739–751. 10.1017/S1461145713001545 24345557 PMC4220740

[B36] Weissmann D. van der Laan S. Underwood M. D. Salvetat N. Cavarec L. Vincent L.. (2016). Region-specific alterations of A-to-I RNA editing of serotonin 2c receptor in the cortex of suicides with major depression. Transl. Psychiatry 6:e878. 10.1038/tp.2016.121 27576167 PMC5022077

[B37] Yamashita T. Kwak S. (2014). The molecular link between inefficient GluA2 Q/R site-RNA editing and TDP-43 pathology in motor neurons of sporadic amyotrophic lateral sclerosis patients. Brain Res. 1584, 28–38. 10.1016/j.brainres.2013.12.011 24355598

[B38] Yoshimoto K. Watanabe Y. Tanaka M. Kimura M. (2012). Serotonin2C receptors in the nucleus accumbens are involved in enhanced alcohol-drinking behavior. Eur. J. Neurosci. 35, 1368–1380. 10.1111/j.1460-9568.2012.08037.x 22512261 PMC3490368

[B39] Yoshimoto K. Yayama K. Sorimachi Y. Tani J. Ogata M. Nishimura A.. (1996). Possibility of 5-HT3 receptor involvement in alcohol dependence: a microdialysis study of nucleus accumbens dopamine and serotonin release in rats with chronic alcohol consumption. Alcohol. Clin. Exp. Res. 20, 311A–319A. 10.1111/j.1530-0277.1996.tb01164.x 8986229

